# Social cues can impact complex behavior unconsciously

**DOI:** 10.1038/s41598-020-77646-2

**Published:** 2020-12-03

**Authors:** Christoph Schütz, Iris Güldenpenning, Dirk Koester, Thomas Schack

**Affiliations:** 1 Faculty of Psychology and Sports Science, Bielefeld University, 33615 Bielefeld, Germany; 2 Faculty of Science, Paderborn University, 33098 Paderborn, Germany; 3 Faculty Business and Management, BSP Business School Berlin, 12247 Berlin, Germany; 4 Center for Cognitive Interaction Technology (CITEC), Bielefeld University, 33619 Bielefeld, Germany; 5 Research Institute for Cognition and Robotics (CoR-Lab), Bielefeld University, 33615 Bielefeld, Germany

**Keywords:** Psychology, Human behaviour

## Abstract

In three experiments, we investigated the effect of unconscious social priming on human behavior in a choice reaction time task. Photographs of a basketball player passing a ball to the left/right were used as target stimuli. Participants had to respond to the pass direction either by a whole-body (complex) response or a button-press (simple) response. Visually masked stimuli, showing both a task-relevant cue (pass direction) and a task-irrelevant, social cue (gaze direction), were used as primes. Subliminal social priming was found for kinematic (center of pressure) and chronometric measures (response times): gaze direction in the primes affected responses to the pass direction in the targets. The social priming effect diminished when gaze information was unhelpful or even detrimental to the task. Social priming of a complex behavior does not require awareness or intentionality, indicating automatic processing. Nevertheless, it can be controlled by top-down, strategic processes.

## Introduction

The unconscious influence of external stimuli on human thought and behavior has long been of interest^1–3^ and is still being debated^4,5^. Studied behaviors range from simple responses (single muscle contractions) to complex, whole-body responses (control of numerous muscles and joints). Effects of subliminally presented stimuli (i.e., outside of subjects’ awareness) have been reported for trait and affective judgments in social psychology^6,7^ and for simple categorization tasks in experimental psychology^8,9^ (button-press responses in a choice reaction time task). In contrast, a subliminal influence of social stimuli on complex behavior is still controversial^4,10–13^.

The most common method in experimental psychology to investigate a subliminal influence of stimuli on behavior is masked priming^14^. In this paradigm, a masking stimulus follows a prime stimulus after a short delay of a few tens of milliseconds. The masking pattern is either presented at the same position as the prime or surrounding it. This backward masking^15^ (i.e., the mask following the prime) can prevent all conscious awareness of the prime, which often is assured in an additional task in which participants are asked to categorize the prime stimulus. Signal detection measures^16^ are commonly computed to assess if a prime was processed unconsciously.

In the last two decades, discussions of unconscious processing and automaticity were guided by theoretical frameworks under the general label of ‘two-systems theories’^17,18^. These theories distinguished between two types of mental processes, automatic and controlled. Automatic processes were assumed to not require (1) intention or (2) conscious awareness and to be (3) not controllable but (4) efficient, that is, to require little working memory capacity^19^. In contrast, intentionality, awareness, controllability, and consumption of working memory resources were considered features of controlled processes.

In recent years, reasonable doubt has been raised against these dichotomous frameworks as being too simplistic^20,21^. A consistent alignment of all four processing features, that is, the assumption that all four features are either true or false (while 14 other feature combinations are rare or nonexistent), seems questionable^22^. A considerable number of studies found misalignments between features (for an overview, see^22^), for example, processes which were unconscious but controllable^23^, suggesting that a gradual perspective on automaticity might be more appropriate.

Here, we ask whether a task-irrelevant but socially relevant cue, human gaze direction, can affect a complex, whole-body response automatically. In a series of three experiments, we investigated the following features of automaticity: need for conscious awareness, intentionality, and controllability.

Gaze direction is considered an important source of social information for interactions among partners^24^, for example, to signal turn taking in conversations^25^ or to establish joint attention^26^. Gaze direction is also an important cue in sports scenarios^27–29^, for example, in basketball. Here, gaze direction can signal the upcoming pass direction of an opponent. However, an opponent can also orient gaze direction away from the pass direction to deceive a defending player (so-called head fake). In such situations, a purely automatic processing of gaze direction by the defender would come with disadvantages in response time and accuracy (cf.^27^).

Several studies investigated intentionality and controllability of gaze processing with conscious awareness in a categorization or detection task. Supraliminal, centrally presented gaze primes^30–32^ were used to direct attention towards peripheral targets. Gaze direction was processed even if the gaze was uninformative^30,32^ or detrimental^31^ to the task, which indicates that the processing of supraliminal gaze cues is initiated without intention and is not controllable (i.e., cannot be suppressed). These features correspond well with the defining characteristics of an automatic process. However, the findings cannot be generalized to subliminal processing.

Only a limited number of studies so far addressed gaze processing without conscious awareness^33–35^. These studies also used a categorization task with centrally presented gaze primes and peripheral targets, but mixed supraliminal and subliminal primes. Supraliminal gaze primes affected response times even when detrimental to the task. In contrast, subliminal gaze primes only affected response times when presented in the context of predictive, supraliminal primes. This indicates that top-down, intentional processes that required awareness of the predictive stimuli were executed^23^. These findings confirm that supraliminal gaze in a simple, button-press task is processed without intention and is not controllable, whereas subliminal gaze processing requires top-down intentionality. Thus, subliminal gaze processing appears to be less automatic than supraliminal processing.

Interestingly, other studies reported social priming without awareness and intentionality: subliminal face (not gaze) primes were processed without predictive, supraliminal primes^36,37^. In these studies, however, target pictures (as well as prime pictures) were faces that had to be categorized by gender. As the target pictures were task-relevant, face stimuli were presumably included into participants’ task set and were used for the intentional, top-down creation of action-trigger conditions^23^. These consciously created action-triggers potentially applied to the subliminal primes as well, since primes were similar to the targets. In contrast, gaze primes in previous studies^33–35^ were dissimilar to the categorized target stimuli, but similar to the supraliminal primes. Thus, subliminal gaze primes were only included into the task set when mixed with similar, supraliminal primes.

In the current study, we tested whether gaze primes that were not relevant to the task would be processed automatically, that is, without awareness, intention, and control. We developed a modified version of the masked priming paradigm^27,38^ to measure the effect of gaze direction on complex, whole-body movements in a controlled lab situation with high ecological validity. In contrast to previous studies, we used complex, photorealistic stimuli as both primes and targets.

Participants’ task was to defend against a life-sized basketball opponent (the target picture) passing the ball to the left/right by executing a lateral blocking movement that matched the opponent’s pass direction (complex, whole-body response task). Also, participants were asked to respond to the pass direction with a standard button-press response (simple, button-press task). Before the target picture, a subliminal (forward and backward masked) prime picture of the same basketball opponent was presented. We tested for an unconscious influence of the social cue (gaze) by manipulating the congruency between the gaze direction of the prime and the pass direction of the target. Beyond standard response times (RTs), changes in the center of pressure (CoP) were analyzed to investigate whether complex, whole-body behavior was influenced by the subliminal gaze cues.

We first tested whether task-irrelevant gaze primes could affect a complex motor response without awareness (but with a possible top-down influence; Exp. 1). Second, we asked if subliminal social processing was driven purely bottom-up, subject to top-down influences (e. g., pre-specified action-trigger conditions^23^), or both, by removing predictive gaze cues from the targets (Exp. 2). Third, we tested whether processing of subliminal social cues could be controlled, that is, suppressed by top-down processes if detrimental to the task. To this end, counter-predictive social information was presented in the targets (Exp. 3). We conducted all experiments as a complex, whole-body response task (Exps. 1-3a) and as a simple, button-press task (Exps. 1-3b).

In Experiment 1, gaze direction in the target pictures always matched pass direction (henceforth, *same* pass and gaze direction *within a stimulus* is termed *consistent*). Thus, gaze direction was fully informative but irrelevant to the task. Based on previous reports^8,23^, a subliminal priming effect of the task-irrelevant gaze direction on RT (Exps. 1a,b) and the horizontal displacement of the CoP (henceforth, *CoP priming*; Exp. 1a) was expected, since action-trigger conditions for the gaze could be intentionally created by top-down, strategic processes^23^. For the pass direction, a reliable priming effect was expected as well. Experiment 1 tested if subliminal social cues can influence a complex, whole-body movement.

**Figure 1:**
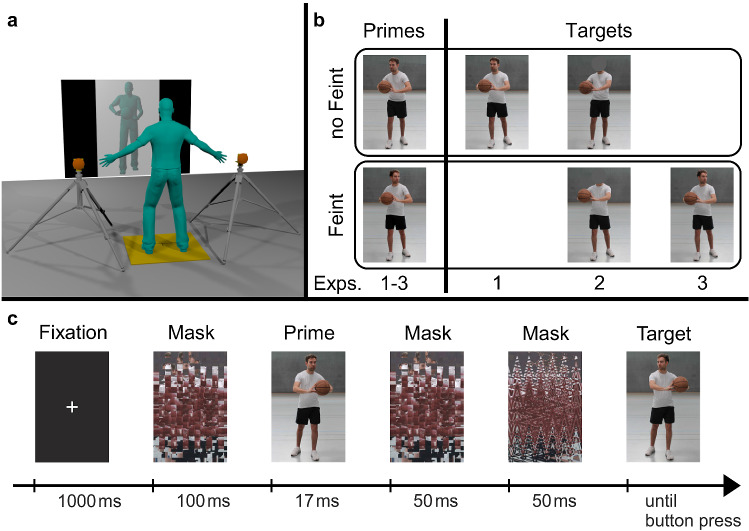
Setup, stimulus examples and procedure of the study. Panel (**a**) shows the setup. Participants were positioned on a force plate facing the projection screen. Response buttons were positioned left and right of the participants in their horizontal plane. Panel (**b**) shows prime-target combinations for all experiments (for a response to the left). Mirrored stimuli were used for a response to the right. In all experiments, half of the trials required a left, the other half a right response. ’No feint’ (*top row*) /’feint’ (*bottom row*) indicates whether the stimulus displayed a head fake or not. As primes, feint and no-feint stimuli were used in all experiments (*first column*). As targets, only no-feint (*second column*), neutral (*third column*), or feint (*fourth column*) stimuli were used for the respective experiments. Panel (**c**) gives the temporal sequence of the stimulus presentation in a single trial (here: for a response to the right). *Notes.* Stimulus pictures for the figure were recreated. Informed consent for publication of identifying images was given. The original stimulus pictures used in the experiment are available on request.

Experiment 2 investigated whether awareness of the social cue (gaze direction) in the target pictures and, thus, top-down, intentional processes are required for subliminal social priming. To this end, the face of the opponent in the target picture was spatially occluded by a gray dot (cf. Fig. 1b, *third column*). Participants were unaware of the presence of gaze cues, since gaze was removed from the target stimuli, while it was still visible in the prime stimuli (but perception was pre-empted by the subliminal presentation). If social priming was independent of a mediating conscious perception, any gaze priming effect in Experiment 1 should be replicated in manner and magnitude. If, however, awareness of the gaze cues and top-down, intentional processes (e. g., pre-specified action-trigger conditions) were required for the subliminal processing of social cues, no or diminished priming effects of gaze would be expected.

Experiment 3 tested whether subliminal social priming can be controlled (i.e., suppressed) by top-down, strategic processes. Here, gaze direction in the target pictures always mismatched pass direction (i.e., gaze and pass direction *within the targets* were *inconsistent*), which resembles a feint or head fake situation in sports^27–29^. As the target pictures were fully visible, participants might try to ignore or suppress the gaze direction, as it was inconsistent with their required movement direction, but were not instructed to do so. If automatic social priming was not amenable to top-down control, comparable gaze priming effects would be expected as in Experiment 2. If social priming was controllable, reduced or no gaze priming effects would be expected.

By comparing the complex, whole-body response task (Exps. 1–3a) and the simple, button-press task (Exps. 1–3b), we tested if the readiness to act in a complex manner is a prerequisite for the supraliminal target stimuli to induce a complex response. Postural sway (i.e., CoP shifts) might be induced by the target stimuli without participants’ awareness (behavioral priming). We asked if the *intention* to respond with a whole-body, sideways movement was necessary to induce postural sway, or if postural sway could be triggered purely by the target stimuli without the intention to execute a complex response. If behavioral priming was partly unconscious^39,40^ (e. g., via mimicry or motor resonance), postural sway would be expected in the simple, button-press task as well. In contrast, ideomotor theories of action planning^41–44^ would predict a controlling influence of intention on behavior and, therefore, no postural sway in Experiments 1–3b.

## Results

### Complex responses (CR)

Based on three exclusion criteria (see Methods), 8.7 % of the trials were removed in Experiment 1a (separated by criterion 1/2/3: 0.0/4.4/4.6 %), 7.5 % in Experiment 2a (0.0/3.4/4.4 %), and 8.1 % in Experiment 3a (0.0/3.7/4.7 %). To test for *congruency* effects (*between prime and target*) on response times (RTs), a 2 (pass congruency) $\times$ 2 (gaze congruency) repeated measures analysis of variance (rmANOVA) was performed.

**Figure 2:**
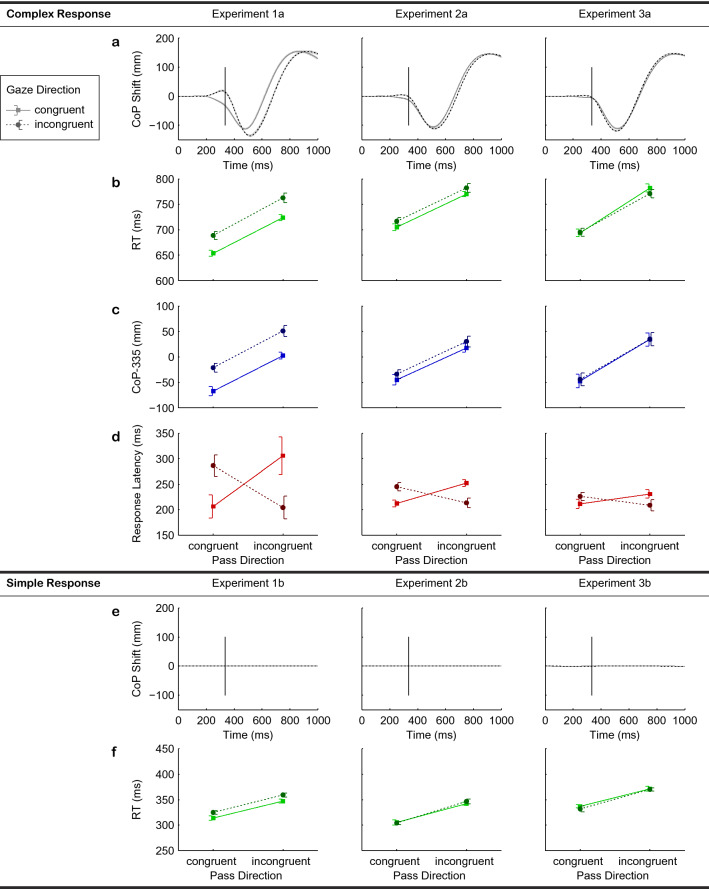
Results of the chronometric and kinematic analyses. All plots depict congruent (*solid lines*) vs. incongruent gaze direction (*dashed lines*). Panels (**a,e**) show lateral movements [shifts of the center of pressure (CoP shifts)], plotted against time. At 0 ms, the prime picture was presented. CoP shifts were rectified; positive values indicate shifts towards the correct response side. For simple responses (**e**), no spatial displacements were detected (yielding flat lines). Panels (**b,f**) show response times (RTs) for whole-body movements (complex response, **b**) and button-press responses (simple response, **f**), plotted against pass congruency. Panel (**c**) depicts the CoP shift 335 ms after prime onset (comparable across exps., see text). Panel (**d**) shows the response latencies (first CoP deviation from rest). *Error bars* represent 95 % (within subject) confidence intervals.

Main effects of pass and gaze congruency in Experiment 1a were significant but the interaction was not (see Table 1). Participants responded faster (–74 ms) to a congruent than incongruent pass direction (see Fig. 2b) and faster (–38 ms) to a congruent than incongruent gaze direction [i.e., gaze in the prime (mis)matching the pass direction in the target].

**Table 1:** Results of the statistical analyses within experiments. *F*- and *t*-values (as indicated with degrees of freedom) with *p*-values (*in brackets*) and effect sizes ($\omega ^2$/Cohen’s *d*). For each dependent variable [response time (RT), center of pressure (CoP-335), response latency], a rmANOVA with the factors *gaze congruency* and *pass congruency* was performed. In the detection task, only *pass direction* of the primes was tested (non significant *d’*-values).

Complex response		Experiment 1a F(1,20)	Experiment 2a F(1,21)	Experiment 3a F(1,21)
*RT*:	gaze	125.36 (<.001); 0.75	19.46 (<.001); 0.30	n. s.
	pass	140.18 (<.001); 0.77	116.82 (<.001); 0.73	123.66 (<.001); 0.74
	gaze $\times$ pass	n. s.	n. s.	12.64 (<.01); 0.12
*CoP-335*:	gaze	126.71 (<.001); 0.75	26.16 (<.001); 0.36	n. s.
	pass	85.58 (<.001); 0.67	46.64 (<.001); 0.51	37.93 (<.001); 0.46
	gaze $\times$ pass	n. s.	n. s.	n. s.
*Resp. Lat.*:	gaze $\times$ pass	17.79 (<.001); 0.17	61.69 (<.001); 0.41	20.35 (<.001); 0.18

Simple response		Experiment 1b F(1,20)	Experiment 2b F(1,21)	Experiment 3b F(1,21)
*RT*:	gaze	60.06 (<.001); 0.58	n. s.	n. s.
	pass	118.24 (<.001); 0.74	118.76 (<.001); 0.73	115.56 (<.001); 0.72
	gaze $\times$ pass	n. s.	n. s.	n. s.

Detection task		Experiment 1 t(20)	Experiment 2 t(21)	Experiment 3 t(21)
*d‘*:	pass	1.42 (.17); 0.31	1.76 (.09); 0.38	0.75 (.46); 0.16

n. s. $$=$$ not significant.

In Experiment 2a (occluded gaze in the targets), again, significant main effects of pass and gaze congruency were obtained (see Table 1). The size of the pass congruency effect (–65 ms) was comparable to Experiment 1a (see Fig. 2b); the size of the gaze congruency effect was smaller (–11 ms). Across experiments, the size of the pass congruency effect did not vary significantly (see Table 2), while the size of the gaze congruency effect did (see Table 2). There was a significant decrease from Experiment 1a to Experiment 2a, $t(41) = -5.88, p < .001, d = 1.79$, and from Experiment 2a to Experiment 3a, $t(42) = -4.24, p < .001, d = 1.28$.

**Table 2:** Results of the statistical analyses across experiments. *F*- and *t*-values (as indicated with degrees of freedom) with *p*-values (*in brackets*) and effect sizes ($\omega ^2$). For each dependent variable [response time (RT), center of pressure (CoP-335), response latency], a rmANOVA with the factors *gaze congruency*, *pass congruency*, and *experiment* (between subject factor) was performed.

Complex response		Experiments 1–3a F(2,62)
*RT*:	gaze $\times$ experiment	49.64 (<.001); 0.27
	pass $\times$ experiment	n. s.
	gaze $\times$ pass $\times$ experiment	n. s.
*CoP-335*:	gaze $\times$ experiment	66.34 (<.001); 0.33
	pass $\times$ experiment	n. s.
	gaze $\times$ pass $\times$ experiment	n. s.
*Resp. Lat.*:	gaze $\times$ pass $\times$ experiment	8.91 (<.001); 0.06

Simple response		Experiments 1–3b F(2,62)
*RT*:	gaze $\times$ experiment	14.17 (<.001); 0.09
	pass $\times$ experiment	n. s.
	gaze $\times$ pass $\times$ experiment	n. s.

n. s. $$=$$ not significant.

In Experiment 3a, the main effect of pass congruency was significant (see Table 1) and similar in size (–83 ms) to Experiment 1a (see Fig. 2b). There was no main effect of gaze congruency, but a significant interaction between pass and gaze congruency. Post-hoc paired-sample *t*-tests (Holm-Bonferroni corrected for family-wise errors) revealed no significant RT difference between the congruent and incongruent gaze direction when pass direction was congruent, $t(21) = -0.28, p = .78, d = .06$. When pass direction was incongruent, there was a significant effect of gaze, $t(21) = 2.63, p = .02, d = .56$, but the direction of the effect was inverted. Participants responded slower (+8 ms) for a congruent than incongruent gaze. The finding, therefore, does not reflect a gaze congruency effect.

To test if statistical results were sensitive to the trial exclusion criteria, all rmANOVAs were redone with all observations (except false responses) included. All findings in Tables 1 and 2 were reproduced, with one notable exception: the interaction between pass and gaze congruency in Experiment 3a was no longer significant, $F(1,21) = 0.387, p = .54, \omega ^2 = -0.01$. Only the interaction was sensitive to the specific trial exclusion criteria (indicating an incidental finding), whereas all remaining results were reliable.

Based on the results of the RT analyses, the CoP shift data was split once by gaze congruency and once by pass congruency. Fig. 2a depicts the CoP shift split by gaze congruency, time-locked to prime onset.

In the congruent gaze conditions in Experiments 1–3a, participants initiated their movement by loading the contralateral leg (i.e., opposite to the response button) resulting in an initial, negative CoP shift (see Fig. 2a, *solid line*). Participants then pushed themselves towards the response button, causing a positive CoP shift. The response button was pressed during the ascending flank of the CoP curve. Similar CoP curves were found in the congruent pass condition in Experiments 1–3a (not depicted).

In the incongruent gaze condition in Experiment 1a, participants initiated their movement by loading the ipsilateral leg, resulting in a small positive CoP shift (see Fig. 2a, *dashed line*) before shifting their weight onto the contralateral leg. Then the movement proceeded as in the congruent gaze condition. The initial positive deflection of the CoP curve was less pronounced in Experiment 2a and absent in Experiment 3a (see Fig. 2a, *dashed lines*). In the incongruent pass condition, the initial positive CoP shift was found in all three experiments and was not attenuated in Experiments 2a and 3a (not depicted).

In Experiments 1–3a, primes with an incongruent pass *and* an incongruent gaze direction resulted in an initial CoP shift in the ipsilateral direction, which was not beneficial for the task. To quantify the size of this erroneous initial CoP shift, the time of the maximum positive deflection for incongruent pass and gaze direction was determined [Exp. 1a: $336 \pm 18\,(SD)$ ms after prime onset; Exp. 2a: $336 \pm 26$ ms, Exp. 3a: $334 \pm 15$ ms]. Therefore, the CoP shift 335 ms after prime onset (henceforth, *CoP-335*) was analyzed.

A 2 (pass congruency) $\times$ 2 (gaze congruency) rmANOVA on the CoP-335 yielded significant main effects of pass and gaze congruency in Experiment 1a (see Table 1). The CoP-335 was more positive (+63 mm) for an incongruent than congruent pass direction (see Fig. 2c) and more positive (+45 mm) for an incongruent than congruent gaze direction. The interaction was not significant. In Experiment 2a, again, only main effects of pass and gaze congruency were found (see Table 1). The pass congruency effect (+63 mm) was comparable to Experiment 1a, the gaze congruency effect was smaller (+12 mm). Across experiments, the size of the pass congruency effect did not vary significantly (see Table 2), while the size of the gaze congruency effect did (see Table 2). There was a significant decrease from Experiment 1a to Experiment 2a, $t(41) = -7.37,\ p < .001,\ d = 2.25$, and from Experiment 2a to Experiment 3a, $t(42) = -3.55,\ p < .001,\ d = 1.07$. In Experiment 3a, only the main effect of pass congruency was significant (see Table 1) and similar in size (+81 mm) to Experiment 1a.

Both incongruent pass and gaze direction resulted in an erroneous initial CoP shift, which might reflect a prime-specific response. A jackknife procedure^45^ was applied to the CoP shift curves over time to measure the onset latency of the movement, with a threshold of 2.5 mm from rest in any direction. Onset latency thus was independent of whether the first CoP shift occurred in a correct or incorrect direction. A retrieval transform^46^ converted the latency scores from the jackknife procedure to latency values viable for a rmANOVA.

A $2\times 2$ rmANOVA was performed to analyze CoP onset latencies. We found neither a main effect of pass congruency nor of gaze congruency in any experiment (1–3a). There was, however, a significant cross-over interaction of both factors in every experiment (1–3a, see Table 1). Response latency was lower (Experiment 1a: –85 ms, Experiment 2a: –36 ms, Experiment 3a: –23 ms) if pass and gaze direction in the prime were consistent (i.e., both congruent or both incongruent), and higher if pass and gaze direction were inconsistent (see Fig. 2d). The size of the cross-over interaction differed across experiments (see Table 2), with a significant decrease from Experiment 1a to Experiment 2a, $t(41) = -2.54,\ p = .02,\ d = 0.78$, and from Experiment 2a to Experiment 3a, $t(42) = -2.88,\ p = .01,\ d = 0.87$. The direction of the initial CoP shift still depended on the congruency to the target’s pass direction: If pass and gaze direction were congruent, the CoP shift was negative, if they were incongruent, it was positive.

### Simple responses (SR)

Based on three exclusion criteria (see Methods), 9.7 % of the trials were removed in Experiment 1b (separated by criterion 1/2/3: 2.6/4.9/3.3 %), 10.8 % in Experiment 2b (3.1/4.8/3.7 %), and 10.0 % in Experiment 3b (2.2/4.7/3.9 %).

A 2 (pass congruency) $\times$ 2 (gaze congruency) rmANOVA on RTs showed significant main effects of pass and of gaze congruency in Experiment 1b but no interaction (see Table 1). Participants responded faster (–34 ms) for a congruent than incongruent pass direction (see Fig. 2f) and faster (–11 ms) for a congruent than incongruent gaze direction. In Experiments 2b and 3b, only the respective main effect of pass congruency was significant (–39 ms and –37 ms, see Table 1). Across experiments, the size of the pass congruency effect did not vary significantly (see Table 2), while the size of the gaze congruency effect did (see Table 2). There was a significant decrease from Experiment 1b to Experiment 2b, $t(41) = -4.17,\ p < .001,\ d = 1.27$, but *not* from Experiment 2b to Experiment 3b, $t(42) = -1.54,\ p = .13,\ d = 0.47$.

CoP shift data was split by gaze congruency and by pass congruency. Fig. 2e depicts the CoP shift split by gaze congruency, time-locked to prime onset. No effect is visible in any of Experiments 1–3b. The same result was found if the CoP shift data was split by pass congruency (not depicted). To verify this result, a $2\times 2$ rmANOVA was conducted on the CoP-335. No main effect and no interaction were significant in any of the experiments (1–3b). For simple responses, the CoP-335 did not depend on pass or gaze congruency.

### Detection tasks

The signal detection measure^16^ *d’* over all participants did not differ from zero in any of the three participant groups (see Table 1). Participants were unable to recognize the primes.

## Discussion

Here, we investigate automatic social priming of complex, whole-body behavior in a highly controlled, experimental setting. Specifically, we test if social priming requires awareness, intentionality, and can be controlled.

For the first time, we demonstrate subliminal response time (RT) priming and center of pressure (CoP) priming for a complex, whole-body response in Experiment 1a, in line with the idea of motor priming^8^. RT results replicate subliminal RT priming effects found for simple (button press) responses in a choice reaction time task (response congruency effect^23,28^): responses after incongruent gaze cues in the prime (i .e., mismatching pass direction in the target) took longer than responses after congruent gaze cues. Moreover, a subliminal priming effect of gaze direction on the CoP was obtained for movement initiation time and for magnitude of displacement, indicating that a task-irrelevant but socially relevant cue (gaze direction) can affect a complex motor response without conscious awareness. These results confirm the proposed unconscious priming of complex behavior by important social cues^10–12^.

Previous work suggests that the conditions for subliminal processing of social primes could be subject to top-down, strategic processes^23,34,36^. Subliminal gaze primes were only processed when presented in the context of informative, supraliminal gaze primes^34,35^. Subliminal face primes were processed when presented in the context of supraliminal face targets in a categorization task^36,37^. These findings indicate that top-down processes (that require conscious awareness of either a target or supraliminal prime that is similar in content to the subliminal prime) were used to define action-trigger conditions^23^. Presumably, these action-triggers were then applied to the subliminal, social stimuli as well. In the present Experiment 1, gaze direction in the target pictures was always *consistent* with pass direction, that is, informative. Thus, despite gaze being irrelevant to the categorization task, participants may have included the social stimulus into their task set.

We therefore eliminated such top-down processes in Experiment 2a by removing conscious awareness of gaze information from the task. To this end, the head was occluded in the *target* pictures. Subliminal social priming (gaze direction) was diminished but reliable. Although priming effects were smaller than in Experiment 1a, the pattern of results was the same, indicating that subliminal social primes were processed purely bottom-up, without intention or relevance to the task. The smaller size of the priming effect in comparison to Experiment 1a, however, implies that the processing of subliminal social primes in Experiment 1a was partially due to top-down, strategic processes, such as action-trigger conditions^23^ that were derived from the consciously processed target pictures.

In Experiment 3a, we tested whether the unconscious processing of detrimental social information can be controlled, that is, suppressed by top-down influences. To this end, the gaze direction in the supraliminal *target* pictures was always *inconsistent* (i.e., counter-predictive) with the task-relevant pass direction. Here, no systematic gaze priming effects could be detected, which indicates that unconscious processing of social information is indeed controllable. Together, our findings show that social cues are processed without conscious awareness and do not require intention (Exp. 2a), which both are characteristics of automaticity. At the same time, social processing is subject to top-down, strategic decisions, and can be amplified in the context of beneficial (Exp. 1a) or suppressed in the context of detrimental (Exp. 3a) social information.

The gaze priming effects gradually decreased across Experiments 1–3a (see Table 2), with consistent gaze information (Exp. 1a), no gaze information (Exp. 2a), and inconsistent gaze information (Exp. 3a) in the target pictures. Neither the amount nor the validity of gaze information can provide an alternative explanation for this effect pattern, as both were identical in Experiments 1a and 3a (gaze direction in Exp. 3a was fully valid but inversed, i.e., inconsistent with pass direction in the targets), yet gaze priming effects differed strongly. Thus, the top-down, strategic explanation of the graded nature of the gaze priming effects is maintained.

Top down, strategic decisions appear to modulate subliminal priming of behavior (consistent with the action-trigger theory^23^). Thus, the present results can help to explain the variability of previous reports of unconscious (social) priming in complex behavior^4,5,13^. Given strategic influences, the framing of any particular study may vary, which can result in different strategic processing and, thus, in variable social priming effects. In light of such top-down control, subliminal social priming effects are unlikely to be as fully automatic as might be expected, for example, by strong embodiment approaches^47^.

The cross-over interaction of pass and gaze direction in movement (CoP) onset latencies (see Fig. 2d) shows that the *(in)consistency* of pass and gaze *within the primes* affected action planning and initiation, independent of the target picture. This effect, which gradually decreased across Experiments 1–3a (see Table 2), suggests that gaze and pass information is extracted separately from the subliminally presented primes, as both information streams slowed down movement onset only when *inconsistent* with one another *within the primes*. This whole-body movement effect is in accordance with button-press results that showed increased RTs for inconsistent gaze and pass directions when evaluating single pictures of basketball players (resembling a feint situation^27^) and with the suggestion of independent processing of prime and target stimuli (rapid chase theory^48,49^).

The cross-over interaction in (CoP) onset latencies is significant in Experiment 3a; the main effect of gaze direction in RT is not. This finding suggests that kinematic measures are more sensitive to subtle priming effects than RT measures alone. Furthermore, the presence of a gaze priming effect in RT in Experiment 2a, in contrast to Experiment 2b, shows that additional insights are gained from an action context. Experiments 1–3b (no action context, evaluative judgments) alone would only replicate the results of Al-Janabi and Finkbeiner^34^, who found that subliminal gaze primes only affected RTs in the context of supraliminal, informative gaze stimuli. From this, Al-Janabi and Finkbeiner concluded that subliminal gaze processing requires intentional, top-down control. In contrast, the present Experiment 2a (with action context) showed that subliminal gaze affected both RTs and CoP without an informative gaze context, which proves that no intentional, top-down control is required for subliminal gaze processing.

By comparing the simple, button press task and the complex, whole-body response task, we could test if the readiness to act in a complex manner is a prerequisite for the target stimuli to induce a complex response. Postural sway (CoP) might be induced by the supraliminal targets without participants’ awareness (behavioral priming). Experiments 1–3b (simple, button-press responses) demonstrated that a mere evaluation of the target pictures yielded no CoP priming. The lack of CoP priming strongly suggests that the *intention* to respond with a complex, whole-body movement is an essential prerequisite for an unconscious behavioral priming; the perception of a social stimulus may not be sufficient in itself. These findings concur with the ideomotor principle^41–44^ and provide further support for theories of action priming^50–52^.

While the task-irrelevant gaze information led to graded priming effects for complex responses (Exps. 1–3a) and for simple responses only in Exp. 1b (consistent gaze and pass direction within targets), pass direction reliably elicited priming effects throughout the experimental series (Exps. 1–3a & 1–3b; see Table 2; Fig. 2b,f). Hence, the variability of the gaze priming effects cannot be explained in terms of group differences as no such variability was observed for the pass priming effects. The stable priming effects of pass congruency together with all groups’ chance performance in the detection task suggest that the experimental manipulations were effective, both regarding the instructions and also in limiting gaze information of the primes to unconscious processing stages.

In conclusion, the results of this highly controlled experimental approach with a high ecological validity suggest that a complex behavioral response cannot be initiated by a supraliminal stimulus without a readiness to act. Once initiated, complex behavior is susceptible to automatic social priming that requires neither awareness nor intention. Nevertheless, the social priming effect can be controlled by top-down, strategic processes. The introduced measurement of whole-body movement kinematics is more sensitive than standard (button-press) RT measurements and provides new insights into the importance of (action) context when studying human behavior.

## Methods

### Power analysis

Before the experiment, a pilot study [$N = 15$ participants, 9 female, mean age $23.0 \pm 2.6\ (SD)$ years, 14 right-handed] was conducted. Stimuli were identical to Experiment 1 (see below). Participants were seated in front of a computer monitor (vertical retraces 60 Hz) and responded by a simple button press. Response time (RT) was measured as the dependent variable by Presentation 16.1 (NeuroBehavioral Systems, Inc., Albany, CA).

An a priori power analysis was calculated using the SPSS (22, IBM Corp., Armonk, NY) MANOVA procedure^53^, based on the means, standard deviations, and correlations of the RTs in the pilot study. To achieve a power above .8 for detecting a main effect of the within subject factor *gaze congruency*, 19 participants were required. Power of the within subject factor *pass congruency* then was above .99.

### Participants

Three groups of students from Bielefeld University participated in one of three experiments each in exchange for course credit (Exp. 1: $N = 21$, 14 female, mean age $27.3 \pm 4.8$ years, 19 right-handed; Exp. 2: $N = 22$, 14 female, mean age $24.9 \pm 4.1$ years, all right-handed; Exp. 3: $N = 22$, 12 female, mean age $27.7 \pm 9.5$ years, 20 right-handed). Each group executed one complex response (CR) task (Experiments 1–3a) and one simple response (SR) task (Experiments 1–3b), respectively. Participants had normal vision, characterized themselves as neurologically healthy and were naïve to the purpose of the study. Participants were recruited from a general university population and had no prior experience in basketball (except for school lessons; for effects of expertise on decision making in sports see^54,55^). Each participant received instructions before participation and gave written informed consent. The study was conducted in accordance with the 1964 Declaration of Helsinki^56^ and approved by the Bielefeld University ethics committee.

### Setup

Participants were positioned on an AMTI BP900-900 force plate (AMTI, Watertown, MA, see Fig. 1a). The position of the participants on the force plate was standardized using the tip of the toes and median plane of the body as reference points. A projection screen (width 244 cm, height 183 cm) was installed 350 cm in front of the participants. Two response buttons were placed on tripods to the left and to the right of the force plate. Each button had a flat surface with a diameter of 14 cm and a convex surface that matched the curvature of a basketball. Button centers were located 195 cm apart and 103.5 cm above ground level, with the flat surface of each button aligned with the tip of the toes and the spherical surface pointing away from the screen (see Fig. 1a).

### Stimuli

Stimuli were created from six photographs of a basketball player (body height 177 cm) passing the ball past the right side of the camera. In three of the photographs gaze direction was consistent with the pass direction (cf. Fig. 1b, *top row*), in the other three photographs it was inconsistent with the pass direction (gaze feints, cf. Fig. 1b, *bottom row*). Photographs were mirrored to create identical stimulus sets for passes to the left, resulting in a total of 12 stimuli. Pre- and post-masks were generated once by scrambling and once by distortion of the original stimuli (see Fig. 1c).

All stimuli ($512\times 768$ pixels, width 122 cm, height 183 cm) were projected on screen to create a life-size depiction of the basketball player. Visual angle was 29.3$^\circ$ in the vertical direction and 19.8$^\circ$ in the horizontal direction. A Canon LV-X6 projector (vertical retraces 60 Hz) and Presentation 16.1 were used for stimulus presentation.

### Procedure

After adopting the predefined, standardized position, participants were asked to respond to the pass direction of the presented target picture (instructed to ’block the passed ball’) as fast and as accurately as possible. In Experiments 1–3a responses were given by a complex, whole-body movement towards the convex response buttons (CR: complex response condition). In Experiments 1–3b responses were given by a simple button press on a hand-held button box (SR: simple response condition). The order of the two experiments in each group was counterbalanced across participants.

Each trial started with the presentation of a fixation cross (1000 ms), followed by one pre-mask (100 ms), the prime (17 ms), two post-masks (50 ms each), and the target (until response, see Fig. 1c). Incorrect responses elicited a visual error feedback. After each response, participants had to return their center of pressure (CoP) to the center of the force plate. Otherwise, the start of the next trial was delayed. Each experiment consisted of 144 trials. Before each experiment, participants performed 24 practice trials.

An experimental session finished with a detection task to test participants’ awareness of the primes. Participants were fully informed about the prime stimuli and asked to respond to the pass direction of the prime pictures by a simple button press on the hand-held button box. No error feedback was provided. The detection task consisted of 72 trials.

### Design

All experiments used a $2\times 2$ factorial design; within-subject factors were *pass congruency* and *gaze congruency*. Congruency for both pass and gaze direction referred to the required response direction, which was defined by the pass direction in the target. Thus, pass/gaze direction in the prime was considered congruent/incongruent if it was the same as/different from the pass direction in the target. For example, both pass and gaze direction in the prime in Fig. 1c are congruent. The full set of stimuli was used as primes in all experiments, thus including both consistent and inconsistent combinations of pass and gaze direction (cf. Fig. 1b, *first column*).

In Experiment 1, target pictures never showed a head fake. That is, only stimuli with consistent pass and gaze direction were used as target stimuli (cf. Fig. 1b, *second column*). Thus, participants might have considered gaze direction a helpful cue for the successful completion of the task, as they were unaware of the existence and the content of the prime stimuli. No instructions regarding gaze information were given in any of the experiments. In Experiment 2, only stimuli with spatially occluded gaze direction were used as target stimuli (cf. Fig. 1b, *third column*). Thus, participants were not aware of gaze information in the stimuli. In Experiment 3, all target pictures showed a head fake. That is, only stimuli with inconsistent pass and gaze direction were used as target stimuli (cf. Fig. 1b, *fourth column*). Thus, participants might have suppressed the distracting gaze information despite no explicit instructions to do so.

Factor combinations were presented in a pseudo-randomized order. In the CR and SR experiments, each factor combination was presented 36 times; in the detection tasks, each factor combination was presented 18 times. Each prime picture and each target picture (and, thus, response direction) was presented equally often. Identical prime and target pictures within the same trial were prevented. A maximum of three subsequent repetitions was allowed for (1) the pass direction in the target, (2) the pass direction in the prime, and (3) each factor combination.

### Measurement

Response buttons were connected to the parallel port of the stimulus presentation computer. Response time (RT) was measured with a precision of one millisecond by the Presentation software. The AMTI force plate was connected to a Vicon MX Ultranet (Vicon Motion Systems, Oxford, UK) with an MX LabControl 64-channel A/D converter (16-bit resolution, 1000 Hz sampling frequency). Ground reaction forces and torques were recorded by Vicon Nexus 1.7.1 on the recording computer. For synchronization purposes, serial port signals were sent from the stimulus presentation computer to the recording computer at the moment of prime presentation. RT data and force plate data were exported to MATLAB (2008a, The MathWorks, Natick, MA) for post-processing.

### Post-processing

From the ground reaction forces and torques, the CoP shift of the participants in the frontal plane was calculated over time. Based on the synchronization signals, 144 sequences (one for each trial) were extracted from the CoP data. Each sequence started with the presentation of the prime and had a duration of 1000 ms. Small fluctuations in the participants’ return positions occurred after each button press. To remove the offset caused by these fluctuations, the CoP shift in the frontal plane at the start of each sequence was subtracted from the sequence data. Sequences were normalized in response direction by inverting the sign of all sequences with leftward responses. Thus, the CoP shift caused by the stimuli was independent of the response direction.

Trials were excluded from the data set if (1) participants gave the wrong response, (2) RT deviated by more than two standard deviations from the average RT, and (3) the root mean squared deviation of the CoP from rest in the first 150 ms after prime presentation differed more than two standard deviations from the average. The last criterion was applied to remove trials in which participants were still adjusting their weight on the force plate (swaying) when the stimulus presentation began.

The remaining CoP shift sequences were averaged for each factor combination, resulting in four sequences over time per participant. The same trial exclusion criteria and averaging procedure were applied to the RT data, resulting in four RT values per participant.

### Analyses

To test for congruency effects in RTs, 2 (pass congruency) $\times$ 2 (gaze congruency) repeated measures analyses of variance (rmANOVAs) were performed. Post-hoc paired-sample *t*-tests (Holm-Bonferroni corrected for family-wise errors) were used as follow-ups for interactions.

To test for congruency effects in CoP, $2\times 2$ rmANOVAs were calculated on the amplitude of the CoP shift 335 ms after prime onset (see Results).

A jackknife procedure^45^ was applied to the CoP shift curves over time to measure movement onset latencies (threshold of 2.5 mm). Latency scores were then converted to latency values viable for $2\times 2$ rmANOVAs by a retrieval transform^46^.

To test whether participants were able to recognize the prime, the signal detection measure^16^ *d’* was calculated based on the error rates for each participant. One level of the response factor (pass direction to the right) was treated as signal, the other level (pass direction to the left) was treated as noise. No corrections based on the log-linear rule were required for any of the participants.

To test for differences in the size of congruency effects and interactions across experiments, 2 (pass congruency) $\times$ 2 (gaze congruency) $\times$ 3 (experiment) rmANOVAs were performed on RTs, CoP shifts, and latency values. Experiment was a between subject factor. Post-hoc unpaired *t*-tests (Holm-Bonferroni corrected for family-wise errors) were used as follow-ups for interactions that included the factor experiment.

To test if the statistical results were sensitive to the (potentially subjective^57^) trial exclusion criteria 2 and 3, all rmANOVAs were performed a second time, with all observations (except false responses) included into the data set.

## Supplementary information

Supplementary Table.
