# Supplementary material for: Social cues can impact complex behavior unconsciously
Source: Sci Rep. 2020 Dec 3;10:21017. doi: 10.1038/s41598-020-77646-2 (PMC7712880; doi:10.1038/s41598-020-77646-2)
Supplement: Supplementary file 1 — Supplementary Table. [file 41598_2020_77646_MOESM1_ESM.pdf]

Supplementary Information for

Social cues can impact complex behavior unconsciously

Christoph Schütz[1,*], **Iris Güldenpenning**[2], **Dirk Koester**[3], **and Thomas Schack**[1,4,5]

[1]Bielefeld University, Faculty of Psychology and Sports Science, Bielefeld, 33615, Germany
[2]Paderborn University, Faculty of Science, Paderborn, 33098, Germany
[3]BSP Business School Berlin, Faculty Business and Management, Berlin, 12247, Germany
[4]Bielefeld University, Cluster of Excellence Cognitive Interaction Technology (CITEC), Bielefeld, 33619, Germany
[5]Bielefeld University, Research Institute for Cognition and Robotics (CoR-Lab), Bielefeld, 33615, Germany
[*]christoph.schuetz@uni-bielefeld.de

Table 1. Mean values (95 % confidence intervals *in brackets*) of temporal and spatial dependent variables for whole-body movements (complex response) and button-press responses (simple response). Response latencies and response times (RTs) are given in ms, center of pressure (CoP-335) in mm. Note: *cc: congruent pass/congruent gaze; ci: congruent pass/incongruent gaze; ic: incongruent pass/congruent gaze; ii: incongruent pass/incongruent gaze*

**Complex Response**	Experiment 1a				Experiment 2a				Experiment 3a			
	cc	ci	ic	ii	cc	ci	ic	ii	cc	ci	ic	ii
RT:	653.5	688.6	723.9	762.5	704.9	716.6	770.1	782.3	693.7	695.1	782.0	771.0
	(6.0)	(8.0)	(5.8)	(9.8)	(6.3)	(7.5)	(5.4)	(8.2)	(7.7)	(8.1)	(7.7)	(8.4)
CoP-335:	-67.2	-21.1	2.4	50.9	-45.0	-33.8	17.4	30.4	-47.4	-44.1	34.3	34.7
	(8.9)	(8.7)	(6.8)	(10.7)	(10.0)	(8.7)	(8.3)	(10.6)	(13.5)	(12.4)	(13.1)	(12.8)
Resp. Lat.:	206.3	286.3	305.8	204.5	212.3	245.2	252.5	213.5	211.5	226.2	230.9	209.0
	(22.5)	(21.0)	(36.9)	(22.4)	(6.6)	(8.3)	(6.8)	(9.2)	(8.7)	(8.0)	(8.3)	(10.8)
**Simple Response**	Experiment 1b				Experiment 2b				Experiment 3b			
	cc	ci	ic	ii	cc	ci	ic	ii	cc	ci	ic	ii
RT:	313.7	324.9	347.2	359.2	305.2	304.0	342.1	346.6	336.7	331.8	371.4	370.1
	(4.3)	(3.7)	(4.2)	(3.7)	(5.4)	(3.1)	(3.1)	(5.0)	(3.0)	(6.1)	(4.7)	(3.3)
